# “When ‘Bad’ is ‘Good’”: Identifying Personal Communication and Sentiment in Drug-Related Tweets

**DOI:** 10.2196/publichealth.6327

**Published:** 2016-10-24

**Authors:** Raminta Daniulaityte, Lu Chen, Francois R Lamy, Robert G Carlson, Krishnaprasad Thirunarayan, Amit Sheth

**Affiliations:** ^1^ Center for Interventions, Treatment, and Addictions Research Department of Population and Public Health Sciences, Boonshoft School of Medicine Wright State University Kettering, OH United States; ^2^ The Ohio Center of Excellence in Knowledge-enabled Computing Department of Computer Science and Engineering Wright State University Dayton, OH United States

**Keywords:** social media, Twitter, cannabis, synthetic cannabinoids, machine learning, sentiment analysis, eDrugTrends

## Abstract

**Background:**

To harness the full potential of social media for epidemiological surveillance of drug abuse trends, the field needs a greater level of automation in processing and analyzing social media content.

**Objectives:**

The objective of the study is to describe the development of supervised machine-learning techniques for the eDrugTrends platform to automatically classify tweets by type/source of communication (personal, official/media, retail) and sentiment (positive, negative, neutral) expressed in cannabis- and synthetic cannabinoid–related tweets.

**Methods:**

Tweets were collected using Twitter streaming Application Programming Interface and filtered through the eDrugTrends platform using keywords related to cannabis, marijuana edibles, marijuana concentrates, and synthetic cannabinoids. After creating coding rules and assessing intercoder reliability, a manually labeled data set (N=4000) was developed by coding several batches of randomly selected subsets of tweets extracted from the pool of 15,623,869 collected by eDrugTrends (May-November 2015). Out of 4000 tweets, 25% (1000/4000) were used to build source classifiers and 75% (3000/4000) were used for sentiment classifiers. Logistic Regression (LR), Naive Bayes (NB), and Support Vector Machines (SVM) were used to train the classifiers. Source classification (n=1000) tested Approach 1 that used short URLs, and Approach 2 where URLs were expanded and included into the bag-of-words analysis. For sentiment classification, Approach 1 used all tweets, regardless of their source/type (n=3000), while Approach 2 applied sentiment classification to personal communication tweets only (2633/3000, 88%). Multiclass and binary classification tasks were examined, and machine-learning sentiment classifier performance was compared with Valence Aware Dictionary for sEntiment Reasoning (VADER), a lexicon and rule-based method. The performance of each classifier was assessed using 5-fold cross validation that calculated average F-scores. One-tailed *t* test was used to determine if differences in F-scores were statistically significant.

**Results:**

In multiclass source classification, the use of expanded URLs did not contribute to significant improvement in classifier performance (0.7972 vs 0.8102 for SVM, *P*=.19). In binary classification, the identification of all source categories improved significantly when unshortened URLs were used, with personal communication tweets benefiting the most (0.8736 vs 0.8200, *P*<.001). In multiclass sentiment classification Approach 1, SVM (0.6723) performed similarly to NB (0.6683) and LR (0.6703). In Approach 2, SVM (0.7062) did not differ from NB (0.6980, *P*=.13) or LR (F=0.6931, *P*=.05), but it was over 40% more accurate than VADER (F=0.5030, *P*<.001). In multiclass task, improvements in sentiment classification (Approach 2 vs Approach 1) did not reach statistical significance (eg, SVM: 0.7062 vs 0.6723, *P*=.052). In binary sentiment classification (positive vs negative), Approach 2 (focus on personal communication tweets only) improved classification results, compared with Approach 1, for LR (0.8752 vs 0.8516, *P*=.04) and SVM (0.8800 vs 0.8557, *P*=.045).

**Conclusions:**

The study provides an example of the use of supervised machine learning methods to categorize cannabis- and synthetic cannabinoid–related tweets with fairly high accuracy. Use of these content analysis tools along with geographic identification capabilities developed by the eDrugTrends platform will provide powerful methods for tracking regional changes in user opinions related to cannabis and synthetic cannabinoids use over time and across different regions.

## Introduction

To design effective prevention, intervention, and policy measures, public health professionals require timely and reliable information on new and emerging drug use practices and trends [1-3]. There is a growing recognition that user-generated content available through Web-based and social media platforms such as Twitter, can be used as a rich data source of unsolicited and unfiltered self-disclosures of substance use and abuse behaviors. Such data could be used to complement and broaden the scope of existing illicit drug use monitoring systems by enhancing their capacity for early identification of new trends [3-6].

Twitter is a microblogging service provider and social network platform that was launched in 2006. Currently, Twitter reports 310 million monthly active users [7] that generate over 500 million tweets per day [8]. Prior research has demonstrated that Twitter can be a useful tool for infodemiology studies of very diverse public health issues [9-12]. Furthermore, the US Twitter population is young and ethnically diverse, which makes analysis of Twitter data particularly suitable for drug abuse surveillance because young adults display the highest rates of drug use behaviors [13].

Because of the high volume of data generated by Twitter users and availability of geographic information, analysis of tweets can help identify geographic and temporal trends [14-17]. The content of tweets, although brief and limited to 140 characters (with some recent relaxation of this limit), can be used to extract information on user attitudes and behaviors related to drug use [15,16,18-22]. Prior research indicates that the ability to separate personal communications from other types of communications such as official/media or retail-related tweets might help reduce the “noise” in social media research and increase the quality of the data for epidemiological surveillance [23,24]. Sentiment analysis is another approach to content analysis of social media data that seeks to understand the opinions (positive, negative, or neutral) expressed regarding selected topics.

Several prior studies used manual coding to classify cannabis, alcohol, and other drug-related tweets by sentiment [15,18,20,21] and source [15,21]. However, such studies, because they relied on manual coding, were limited to the analyses of relatively small samples of tweets. Manual coding is a labor intensive and time consuming process, and its wider application to social media data is human-resource intensive and hence slow, expensive, and difficult in particular for the purpose of identifying emerging trends in real-time. Automation of content analysis tasks would provide powerful tools to examine temporal and geographic trends not just in terms of general tweeting activity [14-17], but also in terms of the types of communications and opinions expressed in such tweets (eg, how the opinions expressed in tweets in relation to emerging cannabis products change over time and vary across different states and regions).

Although several prior studies reported on the development of automated approaches to analyze tobacco and ecigarette-related tweet content [25,26] and to identify adverse effects associated with medical use of pharmaceutical drugs [27,28], there have been very few attempts to apply automated content analysis techniques to analyze drug abuse–related tweets [29]. This lack of research is partially related to the fact that drug-related content adds another layer of ambiguity and difficulty in the development of automated techniques because of pervasive use of slang terminology and implied meanings [30,31]. For example, the sentiment lexicon that generally conveys negative meaning in its conventional uses (eg, “bad,” “wasted,” “faded,” “fucked up”) could express positive sentiment when used in drug-related tweets that describe desired effects of getting intoxicated and high (eg, “I wanna mad amounts of blunts and let’s get faded”; “I get fucked up on this shit, I drink lean and smoke dabs every day”). For this domain-specific usage and meanings of sentiment words (where “bad” comes to mean “good,” such as in the case of being “faded” or “fucked up”), traditional approaches that use sentiment lexicons (eg, Valence Aware Dictionary for sEntiment Reasoning (VADER) [32]) may not perform well, and machine learning techniques, trained using manually coded data, could increase the accuracy of sentiment identification in drug-related tweets.

The study builds on interdisciplinary collaboration that combines drug abuse and computer science research to develop eDrugTrends, a highly scalable infoveillence platform for real-time processing of social media data related to cannabis and synthetic cannabinoid use. Development of eDrugTrends platform is based on previous research and infrastructure created by our research team, including Twitris (for analysis of Twitter data) [33-36] and PREDOSE (for analysis of Web forum data) [37-39].

The key goal of this study is to describe the development and performance of machine learning classifiers to automatically identify tweets by the source/type of communication (personal, official/media, retail) and sentiment (positive, negative, neutral) expressed in cannabis- and synthetic cannabinoid–related tweets. Because prior research identified distinct linguistic and sentiment patterns in personal communication tweets compared with tweets generated by organizational entities [15,23], the study also tests an innovative approach that integrates sentiment and source classification to examine sentiment identification in personal communication tweets.

## Methods

### Data Collection

The eDrugTrends platform [14,15] was used to collect and filter Twitter data available through Twitter’s steaming Application Programming Interface. eDrugTrends filters out non-English language tweets and uses keywords and blacklist words to extract tweets of interest. Keywords related to cannabis products (cannabis in general, marijuana edibles, marijuana concentrates) and synthetic cannabinoids were selected using prior research, media publications, and social media discussions of relevant terms [24]. To increase the accuracy of collected tweets, ambiguous slang terms (eg, blunt, spice) were combined with keywords indicating drug usage (eg, smoke/smoked/smoking). In addition, a “blacklist” of words was used to exclude collection of irrelevant tweets (eg, Emily Blunt, pumpkin spice latte) [14,15]. Performance of selected keywords was continuously monitored to identify emerging new uses, contexts, and meanings of slang terminology. The eDrugTrends platform is a real-time data collection system that initiated cannabis- and synthetic cannabinoid–related Twitter data collection in November 2014.

The Wright State University institutional review board reviewed the protocol and determined that the study meets the criteria for Human Subjects Research exemption 4 because it is limited to publicly available tweets. Tweets used as examples were modified slightly to ensure the anonymity of Twitter users who had posted them.

### Manual Coding

Manual coding was conducted to develop a labeled data set to be used as a “gold standard” for machine learning classifiers. First, 3 drug abuse researchers or “domain experts” (RD, FL, RC) conducted preliminary “open” coding [40] of several batches of 200-300 tweets to develop and refine the coding rules for source (Multimedia Appendix 1) and sentiment classification (Multimedia Appendix 2). Next, to assess intercoder reliability, a random subsample of 300 tweets was selected from a batch of 3000 tweets that were randomly extracted from eDrugTrends database of tweets collected between May and July of 2016. Reliability subsample was coded independently by the first and third authors using QDA Miner [41]. Krippendorff’s Alpha statistic was used to assess intercoder reliability [42]. Coding of personal communication (K Alpha = 0.84) and media-related communication (K Alpha = 0.83) tweets had substantial agreement, while agreement was moderate for retail-related tweets (K Alpha = 0.64). Coding of positive (K Alpha = 0.69) and negative sentiment (K Alpha = 0.68) had an adequate level of agreement. However, coding of neutral/unidentified category of tweets achieved a lower level of intercoder agreement (K Alpha= 0.49), which could be explained by the fact that this category was a more amorphous and eclectic group.

Development of the manually labeled data set involved several phases of coding conducted by the first and third authors. To obtain a more balanced dataset, less common categories (eg, negative or retail-related tweets) were purposefully oversampled (for more details, see Multimedia Appendix 3). Oversampling of underrepresented categories is important in order to obtain a more balanced data set for development of machine learning classifiers, given that significant under sampling of a certain category in the training data can directly impact the quality of classification [26]. To reach a sample size of 4000 tweets for the manually labeled data set for machine learning, more than 8000 tweets were manually reviewed and filtered using QDA Miner [41]. The tweets for manual coding were extracted from the pool of 15,623,869 tweets that were collected by eDrugTrends between May and November 2015.

The sample of 4,000 manually labeled tweets was split into two subsamples–1000 were used to train source classifier, and 3000 were allocated for sentiment classification. Information on the manually labeled tweet numbers by category for each subsample is provided in Multimedia Appendix 4.

### Machine Learning

Because the study aimed to integrate source and sentiment classification by focusing on sentiment in personal communication tweets only, source classification can be seen as a preprocessing step that is done before sentiment classification. First, 1000 tweets were used to train a source classifier (Multimedia Appendix 4). Next, for the remaining 3000 tweets (Multimedia Appendix 4), the source classifier is applied to filter out the media- and retail-related tweets, and then train the sentiment classifiers using only the personal communication tweets.

### Source Classification Models

Development of source classifiers focused only on tweets with URLs. Because all media- and retail-related tweets contained URLs, tweets without URLs could be automatically classified as belonging to the personal communication category. To select 1000 tweets with URLs for source classifier, approximately equal numbers of tweets were randomly sampled from each category–330 official/media-related, 340 retail-related, and 330 tweets that contain URLs from personal communication.

Summary information about the machine learning classification models used in the study is presented in Textbox 1. Source classification tested 2 approaches: Approach 1 used short URLs as they appear in tweets, and Approach 2 expanded URLs to their original version and used unigrams and bigrams obtained from unshortened URLs as features in machine learning (Textbox 1 A). Twitter automatically shortens all links to save character space [43], and such shortened links typically do not contain identifiable words. In contrast, expanded URLs frequently contain useful information that could help improve tweet classification accuracy. Examples of commonly occulting words identified in expanded URLs are presented in Multimedia Appendix 5.

First, performance of source classifiers was assessed for multiclass classification (media, retail, personal). Next, the best performing machine learning algorithm in multiclass classification was selected to assess 3 binary classification tasks: (1) media versus the remaining tweets, (2) retail versus the remaining tweets, and (3) personal communication tweets versus the remaining tweets (Textbox 1 A).

### Sentiment Classification Models

Sentiment classification tested 2 approaches: Approach 1 applied sentiment classification to all tweets, regardless of their source/type, using all 3000 manually labeled tweets (1292 positive, 921 negative, 787 neutral/unidentifiable), and Approach 2 applied sentiment classification to tweets identified as personal communications only, excluding retail and media-related tweets. For this approach, the sample of 3000 tweets was first processed using the best performing source classifier (developed for this study) to identify personal communication tweets, which resulted in a sample of 2633 tweets (Textbox 1 B). The sample of 2633 tweets contained 1157 that were manually labeled as positive, 850 negative, and 626 neutral/unidentifiable. (Note that these numbers are different from the information presented in Multimedia Appendix 4 because extraction of 2633 personal communication tweets was performed using source classifier, while Multimedia Appendix 4 information is based on manual coding).

Performance of sentiment classifiers was examined for multiclass (positive, negative, neutral) and for binary classification tasks. Binary classification focused on positive versus negative tweets to examine how well sentiment classifiers performed on reliable categories (as determined by reliability assessment), excluding neutral/unidentifiable group that reached a low level of agreement among human coders. To test Approach 1 (all tweets, regardless of source/type), binary classification used a data set of 2213 tweets that was obtained after removing 787 neutral tweets from the sample of 3000. To test Approach 2 (personal communication tweets only), binary classification used a dataset of 2007 tweets that was obtained after removing 626 neutral/unidentifiable tweets from the sample of 2633 (Textbox 1 B).

In addition, the study used a lexicon and rule-based method VADER that was developed for the analysis of social media texts [32] to classify manually labeled tweet sample allocated for sentiment analysis (N=3000). VADER performance in classifying manually annotated tweets was compared with the accuracy of machine learning classifiers using a one-tailed *t* test statistic.

**Summary information on classification models tested for tweet classification by source/type and sentiment.**

**A. Classification by source/type**

**Approach 1**: using all tweets, regardless of their source/type

  • Multiclass classification [logistic regression (LR), naive bayes (NB), support vector machines (SVM)]:

      ° Personal versus media versus retail (n=1000)

  • Binary classification (using classifier that showed the best results in multiclass classification):

      ° Personal versus the rest (n=1000)

      ° Retail versus the rest (n=1000)

      ° Media versus the rest (n=1000)

**Approach 2**: using expanded URLs

  • Multiclass classification (LR, NB, SVM):

      ° Personal versus media versus retail (N=1000)

  • Binary Classification (using classifier that showed the best results in multiclass classification):

      ° Personal versus the rest (n=1000)

      ° Retail versus the rest (n=1000)

      ° Media versus the rest (n=1000)

**B. Classification by sentiment**

**Approach 1**: using all tweets, regardless of their source/type

  • Multiclass classification (LR, NB, SVM):

      ° Positive versus negative versus neutral/unknown (N=3000)

  • Binary Classification (LR, NB, SVM):

      ° Positive versus negative (N=2213; neutral excluded)

**Approach 2**: using personal communication tweets only

  • Multiclass classification (LR, NB, SVM):

      ° Positive versus negative versus neutral/unknown (N=2633)

  • Binary Classification (LR, NB, SVM):

      ° Positive versus negative (N=2007; neutral/unknown excluded)

### Building and Assessment of Machine Learning Classifiers

To build classifiers, the tweets were tokenized and all words were processed to convert uppercase letters to lowercase. Because prior research suggests that stop words and complete forms of words can be useful sentiment indicators, particularly in brief texts such as tweets, stop words were retained, and no stemming was applied [44-46]. Next, all the unigrams and bigrams were collected and chi-square test was applied to select the top 500 unigrams and bigrams with highest chi-square scores as features [47]. For each feature t *(i)*, its tf-idf score was calculated in a tweet d *(j)* as w *(i,j)* = tf *(i,j)* × idf *(i)*. Term frequency tf *(i,j)* is the number of times feature t *(i)* occurs in tweet d *(j)*. Inverse document frequency is calculated as idf *(i)* = log(N/df *(i)*), where N is the total number of tweets in the dataset, and df *(i)* is the number of tweets in which feature t *(i)* occurs. Each tweet is represented as a feature vector, and each entry of the vector is the tf-idf score of that feature in the tweet. Three machine learning classification techniques were tested for each classification model/approach: Logistic Regression (LR), Naive Bayes (NB), and Support Vector Machines (SVM). All three are commonly used classification algorithms that are known to achieve good results on text classification tasks [25,26,48,49].

The performance of each classifier was assessed by 5-fold cross validation, which is a commonly used method for the evaluation of classification algorithms that diminishes the bias in the estimation of classifier performance [50]. This approach uses the entire dataset for both training and testing, and is especially useful when the manually labeled data set is relatively small. In 5-fold cross-validation, the manually labeled data set is randomly partitioned into 5 equal-sized subsets. The cross-validation process is then repeated 5 times (the folds). Each time, a single subset is retained as the validation data for testing the model, and the remaining 4 subsamples are used as training data. The 5 results from the folds are then averaged to produce a single estimation. The study reports the average of the precision, recall, and F-scores calculated by the system on different folds. Precision is defined as the number of correctly classified positive examples divided by the number of examples labeled by the system as positive. Recall, also referred to as sensitivity, is defined as the number of correctly classified positive examples divided by the number of positive examples in the manually coded data. An F-score is a combination (harmonic mean) of precision and recall measures [51]. One-tailed *t* test statistic was used to determine which classifiers performed significantly better (*P*<.05).

## Results

### Source Classification

Source classification (Approach 1) that used short URLs demonstrated good performance (Table 1 A). SVM algorithm applied to multiclass classification task achieved a macro average F-score of 0.7972, which was not significantly higher compared with LR (*P*=.09) or NB (*P*=.27) performance (Table 1 A). Table 1 B shows the performance of source classifier that used expanded URLs when applied to multiclass classification task. SVM showed slightly better improvement in performance in multiclass classification, compared with NB and LR algorithms, reaching 0.8141 precision, 0.8119 recall, and an F-score of 0.8102. However, these differences did not reach a level of statistical significance (Table 1 C).

**Table 1:** Performance of multiclass source classifiers.

	Algorithm	Precision	Recall	F-Score
**A. Approach 1, using short URLs (N=1000)**				
	LR^a^	0.8007	0.7946	0.7938
	NB^b^	0.8023	0.7926	0.7936
	SVM^c^	0.8059	0.7976	0.7972
**B. Approach 2, using the unshortened URLs as features (N=1000)**				
	LR	0.8062	0.8026	0.8013
	NB	0.8005	0.7972	0.7953
	SVM	0.8141	0.8119	0.8102
**C. *P* values calculated using** *t* **test to asses statistical significance of differences in classifier performance (F-scores)**				
	Approach 1	SVM vs LR, *P*=.09; SVM vs NB, *P*=.27		
	Approach 2	SVM vs LR, *P*=.13; SVM vs NB, *P*=.10		
	Approach 1 vs 2	LR1 vs LR2, *P*=.19; NB1 vs NB2, *P*=.47; SVM1 vs SVM2, *P*=.19		

^a^LR: logistic regression.

^b^NB: naive bayes.

^c^SVM: support vector machines.

**Table 2:** Performance of SVM source classifiers on binary classification for each source category.

	Type of classification	Precision	Recall	F-Score
**A. Approach 1, using short URLs (N=1000)**				
	Media	0.8873	0.8278	0.8477
	Retail	0.8723	0.7913	0.8117
	Personal	0.8755	0.7976	0.8200
**B. Approach 2, using unshortened URLs (N=1000)**				
	Media	0.8958	0.8639	0.8769
	Retail	0.8881	0.8155	0.8357
	Personal	0.9020	0.8572	0.8736
**C. *P* values calculated using** *t* **test to asses statistical significance of differences in classifier performance (F-scores)**				
	Approach 1	Personal vs Media, *P*=.094; Personal vs Retail, *P*=.27, Media vs Retail, *P*=.07		
	Approach 2	Personal vs. Media, *P*=.38; Personal vs Retail, *P*=.03^a^; Media vs Retail, *P*=.01^a^		
	Approach 1 vs 2	Personal1 vs. Personal2, *P*=.001^a^**;** Retail1 vs Retail2, *P*=.004^a^; Media1 vs Media2, *P*=.049^a^		

^a^Values that show statistically significant differences.

Performance of both source classification approaches was also assessed on binary classification tasks. Because SVM showed slightly better performance in multiclass classification than NB or LR (although not statistically significant), it was selected for evaluation on 3 binary classification tasks using the 1000 tweets: (1) media-related tweets versus the rest of tweets, (2) retail-related tweets versus the rest of tweets, and (3) personal tweets versus the rest of tweets (Table 2). When using short URLs for binary classification task, identification of media-related tweets showed slightly better precision, recall, and overall F-scores compared with identification of retail or personal communication tweets (Table 2 A), although these differences were not statistically significant (Table 2 C). The identification of all 3 source categories benefited significantly when unshortened URLs were used as features in classification. Improvements in F-scores between Approaches 1 and 2 were significant for all 3 categories (Table 2 C). Identification of the personal communication tweets benefited the most reaching 0.9020 precision, 0.8572 recall, and an F-score of 0.8736, compared with an F-score of 0.8200 when using short URLs (*P*<.001). Furthermore, when Approach 2 was used, identification of media and personal communication tweets showed significantly higher F-scores compared with retail-related tweet identification (Table 2 C).

### Sentiment Classification

For general sentiment classification approach that classified all 3000 tweets regardless of their source, SVM results showed better precision (0.7147) than other machine learning classifiers, but LR achieved better recall (0.6763) (Table 3 A). In overall F-scores, SVM achieved slightly better results (F=0.6723) than other machine learning classifiers, but the differences were not statistically significant (Table 3 C). However, all 3 machine-learning algorithms achieved better results than the lexicon and rule based method VADER. Compared with VADER (F=0.5116), SVM performance was over 30% better, and the difference was statistically significant at *P*<.001 (Table 3 C).

Before sentiment classification Approach 2 could be applied, the sample of 3000 tweets had to be processed to extract personal communication tweets. Because the SVM source classifier with unshortened URLs showed better performance than other classifiers (Table 2), it was used to identify the personal communication tweets (2633) from the sample of 3000. Table 3 B shows evaluation of sentiment classification of personal communication tweets. Compared with Approach 1 (Table 3 A), multiclass sentiment classification of personal communication tweets (Approach 2) showed approximately 3% improvement for NB, 4% improvement for LR, and 5% for SVM classifier, although these increases did not reach a level of statistical significance (Table 3 C). The NB classifier achieved the greatest precision (0.7539), but SVM showed the highest recall scores (0.7021). Overall, the SVM classifier demonstrated slightly better performance than the other 2 machine learning classifiers by achieving an F-score of 0.7062, which was significantly greater compared with LR and NB, but these difference did not reach statistical significance. All 3 machine-learning classifiers achieved better accuracy than VADER. The F-score of SVM was over 40% greater in comparison to VADER performance, and the difference was statistically significant at *P*<.001 (Table 3 C). The most discriminative unigram and bigram features reflect thematic categories pertinent to each source category (Multimedia Appendix 6).

**Table 3:** Performance of multiclass sentiment classifiers (positive, negative, neutral).

	Algorithm	Precision	Recall	F-Score
**A. Approach 1, including all tweets regardless of the source (N=3000)**				
	LR^a^	0.7047	0.6763	0.6703
	NB^b^	0.7101	0.6693	0.6683
	SVM^c^	0.7147	0.6691	0.6723
	VADER^d^	0.5213	0.5261	0.5116
**B. Approach 2, including personal communication tweets only (N=2633)**				
	LR	0.7145	0.6996	0.6931
	NB	0.7539	0.6914	0.6980
	SVM	0.7442	0.7021	0.7062
	VADER	0.5153	0.5211	0.5030
**C. *P* values calculated using** *t* **test to asses statistical significance of differences in classifier performance (F-scores)**				
	Approach 1	SVM vs LR, *P*=.38; SVM vs NB, *P*=.23; SVM vs VADER, *P*<.001^e^		
	Approach 2	SVM vs LR, *P*=.05; SVM vs NB, *P*=.13; SVM vs VADER, *P*<.001^e^		
	Approach 1 vs 2	LR1 vs LR2, *P*=.08; NB1 vs NB2, *P*=.06; SVM1 vs SVM2, *P*=.052		

^a^LR: logistic regression.

^b^NB: naive bayes.

^c^SVM: support vector machines.

^d^VADER: Valence Aware Dictionary for sEntiment Reasoning.

^e^Values that show statistically significant differences.

**Table 4:** Performance of binary sentiment classifiers (positive vs negative).

	Algorithm	Precision	Recall	F-Score
**A. Approach 1, including all tweets regardless of the source (N=2213)**				
	LR^a^	0.8700	0.8495	0.8516
	NB^b^	0.8797	0.8491	0.8540
	SVM^c^	0.8803	0.8513	0.8557
**B. Approach 2, personal communication tweets only (N=2007)**				
	LR	0.8878	0.8728	0.8752
	NB	0.8892	0.8629	0.8666
	SVM	0.8964	0.8757	0.8800
**C. *P* values calculated using** *t* **test to asses statistical significance of differences in classifier performance (F-scores)**				
	Approach 1	SVM vs LR, *P*=.20; SVM vs NB, *P*=.36;		
	Approach 2	SVM vs LR, *P*=.20; SVM vs NB, *P*=.003^d^;		
	Approach 1 vs 2	LR1 vs LR2, *P*=.04^d^; NB1 vs NB2, *P*=.13; SVM1 vs SVM2, *P*=.045^d^		

^a^LR: logistic regression.

^b^NB: naive bayes.

^c^SVM: support vector machines.

^d^Values that show statistically significant differences.

As shown in Table 4 A, for binary sentiment classification (Approach 1), the SVM classifier showed the best precision and recall scores. The SVM algorithm achieved an F-score of 0.8557, which was slightly higher than LR and NB, although the differences were not statistically significant (Table 4 C). When sentiment classification was performed on personal communication tweets only (Table 4 B), LR and SVM performance showed statistically significant improvement in comparison to Approach 1 binary classification task (Table 4 C). The SVM classifier achieved high precision and recall (both of which approached 90%), and an F-score of 0.8800, which was significantly greater in comparison to NB, but not significantly different from LR (Table 4 C). Results of binary classification tasks were not compared with VADER, because the latter still classifies tweets into 3 categories assigning a tweet to a neutral category when it cannot find any sentiment words/patterns.

The most discriminative unigram and bigram features that were identified by chi-square test reflect thematic groups as pertinent to sentiment categories: “want,” “love,” “need” for positive, in contrast to “don’t,” “shit,” “fake” for negative tweets (Multimedia Appendix 7). Our sentiment classifier tended to incorrectly classify tweets that expressed an opposing opinion to negative thoughts or actions related to cannabis use or its legalization. For example, the following tweets were classified as negative by our classifier, although manual coding identified them as conveying positive views toward cannabis: “@GovChristie very ignorant to not see the value of cannabis”; “I think it's ridiculous professional athletes get penalized for smoking a joint....” Humorous and sarcastic tweets were also more difficult to classify correctly by our classifier. For example, the following tweet was coded by domain experts as conveying a positive attitude toward marijuana, but was coded as negative by our machine learning classifier: “Marijuana - side effects may include being happy and consumption of fast food.”

## Discussion

### Principal Findings

The results of this study provide an example of the use of supervised machine learning methods to categorize cannabis- and synthetic cannabinoid–related content on Twitter with fairly high accuracy. To classify tweets by source/type of communication, an SVM algorithm that used expanded URLs produced the best results, in particular as demonstrated by binary classification tasks. For sentiment classification, the SVM algorithm that focused on “personal communication” tweets, in particular classifying positive versus negative tweets only, performed better than a more general approach that included all tweets regardless of the source.

Integration of the 2 dimensions of content analysis tasks—identification of type of communication and sentiment—represents a novel approach. Identification of sentiment in user-generated tweets (personal communications) carries greater relevance for drug abuse epidemiology research than an approach that does not separate personal from media- and retail-related tweets. Use of these content analysis tools along with geographic identification features currently functional in the eDrugTrends platform [14] will provide powerful methods for tracking regional changes in user sentiments related to cannabis and synthetic cannabinoids use over time and across different states or regions.

Overall, our machine learning methods for sentiment classification demonstrated substantially better performance than the lexicon and rule-based method VADER [32]. Prior research has shown that VADER method can achieve an F-score of 0.96 in identifying sentiment when applied to “general” tweets. It is noteworthy that VADER accuracy in classifying tweets in drug use–related domain (where negative words sometime can convey positive and desired experiences) was substantially lower (F=0.51). The accuracy of SVM multiclass sentiment classifier that focused on personal communication tweets only was 40% better in comparison to VADER performance, and the difference was statistically significant at *P*<.001.

Our study demonstrates that content analysis and manual coding of drug-related tweets is not an easy task even for human coders with substantial experience in drug abuse research and qualitative content analysis. This is consistent with prior studies that have reported high level of ambiguity and lack of context as complicating factors in content analysis of tweets [52]. Although our study demonstrates strong performance of machine learning classifiers for automatic classification of tweet content, manual coding will remain an important method necessary for exploration of new domains and improvement of existing automated classification techniques to reflect changes in drug use practices and/or slang terminology. Our experiences developing the labeled data set emphasize the importance of: (1) revealing ambiguities and difficulties encountered when conducing manual coding, and (2) using appropriate metrics to assess intercoder reliability [42].

### Limitations

One of the limitations of our study is that we did not include development of machine learning classification methods to identify relevant and irrelevant tweets (eg, cases were “spice” may refer not to synthetic cannabinoids but to food seasoning). Relevance of extracted data was monitored using appropriate keyword combinations and blacklisted words [15]. We also note the limitations in relation to our ability to identify neutral tweets because they were grouped together with the “unidentifiable” or “difficult to classify” tweets. Until better methods are developed, our future applications of eDrugTrends sentiment analysis tools will take into consideration that neutral/unidentifiable group is a nonreliable category, and will focus on drawing conclusions about positive/negative sentiment tweets only.

Future research will assess performance of these techniques to analyze tweets mentioning other drugs of abuse and will also extend them to automate extraction of more detailed thematic information from drug-related tweets. In addition, because many tweets contain visual information to convey meaning, machine learning–based image classification would add an additional dimension and improve the accuracy of overall tweet content classification. In the future, we will examine the feasibility of separating true neutral tweets from unidentifiable group to improve sentiment analysis.

### Conclusions

This is one of the first studies to report successful development of automated content classification tools to analyze recreational drug use–related tweets. These tools, as a part of eDrugTrends platform, will help advance the field’s technological and methodological capabilities to harness social media sources for drug abuse surveillance research. Our future deployment of the eDrugTrends platform will generate data on emerging regional and temporal trends and inform more timely interventions and policy responses to changes in cannabis and synthetic cannabinoid use practices.

## Appendix

Multimedia Appendix 1 Source classification: coding guidelines used to manually annotate tweets as personal, retail-, and media-related communications.

Multimedia Appendix 2 Sentiment classification: coding guidelines used to manually annotate tweets as expressing positive, negative, or neutral/unidentifiable sentiment.

Multimedia Appendix 3 Description of the development of manually labeled data set.

Multimedia Appendix 4 Information about the manually labeled tweets included in subsets to train source and sentiment classifiers.

Multimedia Appendix 5 Commonly occurring words in unshortened URLs by source/type category.

Multimedia Appendix 6 Top 10 most discriminative unigram and bigram features for source classification.

Multimedia Appendix 7 Top 10 most discriminative unigram and bigram features for sentiment classification.

## Abbreviations

LR: logistic regression
NB: naive bayes
SVM: support vector machines
VADER: Valence Aware Dictionary for sEntiment Reasoning
