# Supplementary material for: “When ‘Bad’ is ‘Good’”: Identifying Personal Communication and Sentiment in Drug-Related Tweets
Source: JMIR Public Health Surveill. 2016 Oct 24;2(2):e162. doi: 10.2196/publichealth.6327 (PMC5099500; doi:10.2196/publichealth.6327)
Supplement: Multimedia Appendix 1 [file publichealth_v2i2e162_app1.pdf]

**Category**	**Description**	**Tweet examples**
Personal communication	Tweets posted by individual users; express personal experiences, opinions and thoughts	“At work, munching on edibles”<br><br>“Going to rip a few dabs”
Media/official communication tweets	News reports, scientific study results, and other information shared by official sources, including government agencies, non-profits, and media sources.	“Marijuana Legalization In Ohio 2015: Voters Could Decide To Legalize Weed In November As Measure Moves Forward http://t.co/cguOscOxZ7”
Retail-related tweets	Information shared by retail organizations about product and service promotion	“It's CBD Sunday! 10% off all CBD flower, tincture, edibles and concentrates!”
