# Supplementary material for: “When ‘Bad’ is ‘Good’”: Identifying Personal Communication and Sentiment in Drug-Related Tweets
Source: JMIR Public Health Surveill. 2016 Oct 24;2(2):e162. doi: 10.2196/publichealth.6327 (PMC5099500; doi:10.2196/publichealth.6327)
Supplement: Multimedia Appendix 2 [file publichealth_v2i2e162_app2.pdf]

Category	Description/Thematic Groups	Tweets Examples
**Positive**	• Expression of liking, approval (like, love, support)	"I love dabs"
	• Positive qualities of drugs or people who use them	"Yea, I remember…edibles are a lot better"
	• Indication of drug usage, intentions to use, buy, or share drugs; encourage others to us	"I need some ganja"<br>"Just found 50 bucks, going to buy dabs, you wanna join? Hmu"<br>"Vendor Day! Free candy and information on infused edibles. #edibles http://t.co/GV8XeLLf9"
	• Positive/desirable effects	"Tried my first dab Tuesday night. Best sleep I've had in a while"<br>"Study: #Cannabis Users Have Lower Incidence of Bladder Cancer. http://t.co/XkpQiR2dfW"
**Negative**	• Expression of disliking, disapproval (don't like, hate)	"I hate edibles"
	• Negative characteristics of drugs and/or people who use them	"Girls who smoke spice are gross asf"
	• Indicate non-usage, plans to quit using, or discourage others from using	"I didn't smoke no spliff on 420"<br>"Don't ever smoke synthetic pot u guys"
	• Negative/undesirable effects	"Dabs are too much, I'm fucking dying"<br>"My girl said that the spliff takes the soul from me"<br>"Surge in synthetic pot use linked to rise in violent crime http://t.co/hjD4yylxE"
**Neutral/ unidentifiable**	• Tweets that report factual information/no opinion	"New York Begins Accepting Medical Marijuana Applications http://t.co/gbs0t7m1F"
	• Tweets as questions	"What do dabs feel like?"
	• Too ambiguous/unclear	"[NAME] enjoy your porridge you'll be passed around like a spliff"
	• Not enough textual information (Tweets with a link to visual content that helps explain the meaning were coded as neutral because visual content was not analyzed).	"Me, when I'm high on edibles: http link"
