# Supplementary material for: “When ‘Bad’ is ‘Good’”: Identifying Personal Communication and Sentiment in Drug-Related Tweets
Source: JMIR Public Health Surveill. 2016 Oct 24;2(2):e162. doi: 10.2196/publichealth.6327 (PMC5099500; doi:10.2196/publichealth.6327)
Supplement: Multimedia Appendix 3 [file publichealth_v2i2e162_app3.pdf]

After manually coding the first batch of 1,000 randomly selected tweets that were extracted from the eDrugTrends database, it was observed that the random sample contained very few tweets that conveyed negative sentiment or were related to retail communications, for example.

To develop a more balanced manually labeled data set, the first and third authors went through several phases of sample building, each of which included the following steps:

1) Using the eDrugTrends platform to extract about 2,000-3,000 tweets (related to cannabis in general, synthetic cannabinoids, marijuana edibles, or marijuana concentrates);
2) Uploading the extracted sample to QDA Miner [41];
3) Reviewing a random subset of about 800-1,000 tweets, on average, using QDA Miner's data filtering features and coding by source and sentiment;
4) Extracting coded tweets and adding to the manually labeled data set; when selecting tweets to add to the manually labeled data set, an attempt was made to exclude duplicate tweets (e.g., re-tweets that that contained identical textual content) and to selectively oversample less commonly encountered categories such negative tweets or retail-related tweets.

These steps were repeated with seven samples of tweets extracted separately for cannabis, marijuana edibles, marijuana concentrates and synthetic cannabinoids.
