# Supplementary material for: “When ‘Bad’ is ‘Good’”: Identifying Personal Communication and Sentiment in Drug-Related Tweets
Source: JMIR Public Health Surveill. 2016 Oct 24;2(2):e162. doi: 10.2196/publichealth.6327 (PMC5099500; doi:10.2196/publichealth.6327)
Supplement: Multimedia Appendix 4 [file publichealth_v2i2e162_app4.pdf]

| A. Manually Labeled Tweets Used to Train Source Classification (N=1,000) | | | |
|---|---|---|---|
| Personal:<br>330 (33%) | Media:<br>330 (33%) | Retail:<br>340 (34%) | Total:<br>1,000 (100%) |
| **B. Manually Labeled Tweets Used to Train Sentiment Classification (N=3,000)** | | | |
| ***Personal communication tweets only:*** | | | |
| Positive:<br>1,139 (45%) | Negative:<br>794 (31%) | Neutral/Unknown:<br>606 (24%) | Total:<br>2,539 (100%) |
| ***Media related tweets only:*** | | | |
| Positive:<br>139(32%) | Negative:<br>127 (29%) | Neutral/Unknown:<br>174 (39%) | Total:<br>440 (100%) |
| ***Retail related tweets only:*** | | | |
| Positive:<br>14 (67%) | Negative:<br>0 | Neutral/Unknown:<br>7 (33%) | Total:<br>21 (100%) |
| ***All tweets:*** | | | |
| Positive:<br>1,292 (43%) | Negative:<br>921 (31%) | Neutral/Unknown:<br>787 (26%) | Total:<br>3,000 (100%) |
