# Supplementary material for: “When ‘Bad’ is ‘Good’”: Identifying Personal Communication and Sentiment in Drug-Related Tweets
Source: JMIR Public Health Surveill. 2016 Oct 24;2(2):e162. doi: 10.2196/publichealth.6327 (PMC5099500; doi:10.2196/publichealth.6327)
Supplement: Multimedia Appendix 5 [file publichealth_v2i2e162_app5.pdf]

**Tweet Type /source**	**Examples of Common Words Appearing in Expanded URLs**
Personal communication	Twitter, Instagram<br><br>(Personal communication tweets include links to Twitter or other social media status updates)
Media-related communication	news, media, forbes, hightimes, latimes, nytimes, Fox10
Retail-related communication	RefillMyMeds, MMJEdibles, ColoradoMMJ
