# Supplementary material for: “When ‘Bad’ is ‘Good’”: Identifying Personal Communication and Sentiment in Drug-Related Tweets
Source: JMIR Public Health Surveill. 2016 Oct 24;2(2):e162. doi: 10.2196/publichealth.6327 (PMC5099500; doi:10.2196/publichealth.6327)
Supplement: Multimedia Appendix 6 [file publichealth_v2i2e162_app6.pdf]

**Category**	**Unigram and Bigram Features**
Personal	keif, when, my, trapgrampa, kick, me, time, smoke, shop, night
	when the, kick in, brownie time, rt trapgrampa, trapgrampa pot, pot brownie, edibles kick, me like, freshair in, got me
Media-related	raid, santa, police, ana, video, shop, eat, shows, cops, officers
	santa ana, pot shop, ana police, shop raid, eating edibles, medical marijuana, high times, shows officers, officers eating, video shows
Retail-related	our, today, all, we, Monday, free, concentrates, chocolate, sale, delivery
	off all, edibles today, we have, on all, our edibles, members save, save on, pot shop, today like, munchie monday
