# Supplementary material for: “When ‘Bad’ is ‘Good’”: Identifying Personal Communication and Sentiment in Drug-Related Tweets
Source: JMIR Public Health Surveill. 2016 Oct 24;2(2):e162. doi: 10.2196/publichealth.6327 (PMC5099500; doi:10.2196/publichealth.6327)
Supplement: Multimedia Appendix 7 [file publichealth_v2i2e162_app7.pdf]

Category	Unigram and Bigram Features
Positive	want, love, need, nice, roll, light, food, bed, good, happy
	just want, want blunt, smoke blunt, you smoke, need blunt, want to, love dabs, all want, want edibles, dabs nice
Neutral	smoke, calli, fuckjcampos, snoop, brownie, kick, dogg, question, robertkazinsky, thatgyaltoni
	calli edibles, fuckjcampos when, kick in, edibles hit, edibles kick, snoop dogg, when your, pot brownie, what about, the pot
Negative	don't, shit, fake, why, if, fuck, niggas, stop, fucking, dangerous
	don’t smoke, that shit, if you, fake weed, still smoke, would you, why would, stop smoking, never smoke, hate edibles
